# Unveiling the intricate interplay: Exploring biological bridges between renal ischemia-reperfusion injury and T cell-mediated immune rejection in kidney transplantation

**DOI:** 10.1371/journal.pone.0311661

**Published:** 2024-12-23

**Authors:** Xinyi Xia, Xinrui Fan, Shan Jiang, Yuhan Liao, Yang Sun

**Affiliations:** 1 Department of Cardiology, Union Hospital, Huazhong University of Science and Technology and Key Laboratory of Biological Targeted Therapy of the Ministry of Education, Tongji Medical College, Wuhan, China; 2 Faculty of Psychology, Sleep and NeuroImaging Center, Southwest University, Chongqing, China; 3 Department of Nephrology, Union Hospital, Tongji Medical College, Huazhong University of Science and Technology, Wuhan, China; 4 Department of Medical Records Management and Statistics, Union Hospital, Tongji Medical College, Huazhong University of Science and Technology, Wuhan, China; Rutgers: Rutgers The State University of New Jersey, UNITED STATES OF AMERICA

## Abstract

Although the link between ischemia-reperfusion injury (IRI) and T cell-mediated rejection (TCMR) in kidney transplantation (KT) is well known, the mechanism remains unclear. We investigated essential genes and biological processes involved in interactions between IRI and TCMR. **Methods:** Renal IRI and TCMR datasets were obtained from the Gene Expression Omnibus database. IRI and TCMR co-expression networks were built using weighted gene co-expression network analysis, and essential modules were identified to acquire shared genes and conduct functional enrichment analysis. Shared genes were used for TCMR consensus clustering, differentially expressed genes (DEGs) were identified, and gene set enrichment analysis (GSEA) was conducted. Three machine learning algorithms screened for hub genes, which underwent miRNA prediction and transcription factor analysis. Hub gene expression was verified, and survival analysis was performed using Kaplan–Meier curves. **Results:** IRI and TCMR shared 84 genes. Functional enrichment analysis revealed that inflammation played a significant role. Based on shared genes, TCMR was divided into two clusters. GSEA revealed that graft rejection-related pathways varied between the two clusters. TCMR hub genes, guanylate-binding protein 1 (GBP1) and CD69, showed increased expression. Decreased survival rates were found in patients who had undergone KT and had high GBP1 and CD69 levels. **Conclusions:** The study demonstrates that renal IRI has a potential role in renal TCMR and the pathogenic pathways are potentially inflammation-related.

## Introduction

Kidney transplantation (KT) is the primary therapeutic choice for patients with end-stage kidney disease to improve survival and quality of life. However, improving graft tolerance and extending graft survival remain the major challenges in KT. Acute T cell-mediated rejection (TCMR) is a severe clinical concern during the early phase of KT. TCMR is less concerning than antibody-mediated immune rejection because it is curable and uncommon six years post-transplantation [1]. However, Ho *et al*. discovered a significant prevalence of persistent TCMR in grafts [2]. Meanwhile, Mizera *et al*. suggested that chronic active TCMR is a contributor to KT graft loss [3], and TCMR was shown to be associated with kidney graft fibrosis [4]. In addition, graft loss has been linked to TCMR in young patients who have undergone KT [1]. According to Rampersad *et al*., the first occurrence of TCMR is related to a higher risk of all-cause graft loss [5]. Moreover, a prospective study showed that the combination of delayed graft function and TCMR significantly contributed to graft loss [6]. Therefore, investigating the mechanisms of TCMR is critical.

Ischemia-reperfusion injury (IRI) is an unavoidable and harmful condition caused by graft acquisition, preservation, and blood supply recovery during KT. Renal IRI is closely linked to a decline in graft survival and the emergence of acute rejection (AR) [7]. Significant advances have been made in understanding the pathogenic mechanisms of IRI. Excessive reactive oxygen species cause oxidative phosphorylation in renal IRI. IRI also activates the complement system in the kidneys [8]. Several mechanisms of cellular demise, encompassing ferroptosis, pyroptosis, and necroptosis, participate in the pathogenesis of IRI. Furthermore, neutrophil recruitment is an important inflammatory mechanism in acute kidney injury (AKI) caused by IRI [9].

**Fig 1:**
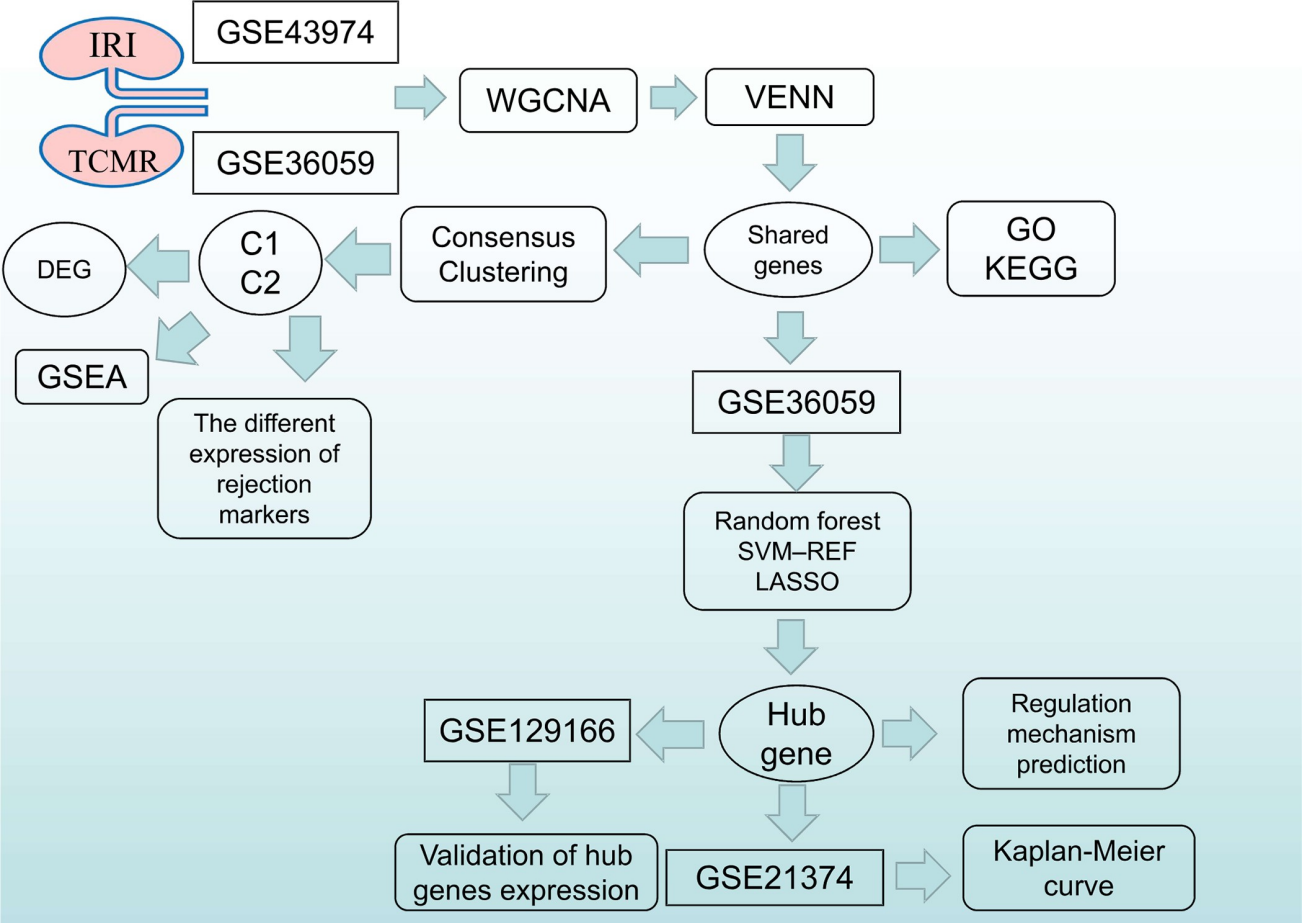
Research flowchart. (WGCNA, weighted correlation network analysis; GO, Gene Ontology; KEGG, Kyoto Encyclopedia of Genes and Genomes; DEGs, differentially expressed genes; GSEA, Gene set enrichment analysis; SVM-RFE, support vector machine recursive feature elimination; LASSO, least absolute shrinkage and selection operator).

The potential mechanisms underlying IRI and TCMR are related to the stimulation of T cell migration, production of inflammatory molecules such as damage-associated molecular patterns, and tumor necrosis factor (TNF) [7]. However, further research into the precise mechanisms is required to advance efforts to increase graft survival in patients undergoing KT. Exploring the probable mechanisms of IRI and TCMR will grant a better understanding of the incidence of KT rejection and guide research in KT therapy and prevention. Thus, we used a bioinformatics approach to explore the potential mechanisms and hub genes involved in IRI and TCMR. The flowchart of the research is shown in Fig 1.

## Methods

### Dataset acquisition

Renal IRI and TCMR datasets were collected from the Gene Expression Omnibus database; the details of these datasets are shown in Table 1.

**Table 1:** Gene expression omnibus datasets.

	GSE number	Platform	Sample	Organism	Disease	Group
**1**	GSE43974	GPL10558	203 patients and 188 controls	Homo sapiens	IRI	Discovery
**2**	GSE36059	GPL570	35 patients and 281 controls	Homo sapiens	TCMR	Discovery
**3**	GSE129166	GPL570	13 patients and 60 controls	Homo sapiens	TCMR	Validation
**4**	GSE21374	GPL570	282 patients who underwent KT	Homo sapiens	KT	Validation

### Weighted Correlation Network Analysis (WGCNA)

WGCNA is a systems biology approach for elucidating gene connection patterns. To exclude outlier data, the expression matrix was first checked for missing values before clustering genes with comparable gene expression patterns. Second, a soft threshold β was chosen based on scale-free network requirements, and the adjacency matrix was turned into a topological overlap matrix for hierarchical clustering. A dynamic shearing method was used to identify modules, and comparable modules were clustered and merged. The minimum number of genes in the module was set to 50, and modules with an Eigen factor greater than 0.75 were merged. Finally, the disease correlation, module membership, and gene significance of each module were calculated, and the key modules with the highest correlations were chosen for further analysis [10].

### Identification of Differentially Expressed Genes (DEGs)

The R software (4.2.2) "limma" package was used to identify different subtypes of TCMR DEGs. Genes with adjusted *p*-values < 0.05 and a log fold change (logFC) > 0.5 were identified as DEGs.

### Functional analysis of shared genes

The protein-protein interaction (PPI) network was built using the STRING (v11.5) database platform. Gene ontology (GO) and Kyoto Encyclopedia of Genes and Genomes (KEGG) pathway enrichment analysis were performed for shared genes. GO analysis provides information on biological processes, molecular functions, and cellular components of genes. KEGG pathway enrichment analysis provides insights into the associated pathways in which the shared genes are involved. Gene set enrichment analysis (GSEA) was used to analyze the different potential biological mechanisms between the two TCMR clusters. An adjusted-*p* < 0.05 was considered significant.

### Consensus clustering

The "ConcensusClusterPlus" package was used to perform consensus clustering. The number of clusters was set between 2 and 6, and the process was repeated 50 times to extract 80% of the total sample using clusterAlg = "hc" and distance = "pearson" [11].

### Screening of hub genes

The least absolute shrinkage and selection operator (LASSO), support vector machine recursive feature elimination (SVM-RFE), and random forest analysis were performed to identify hub genes. LASSO is a regression analysis method that permits variable screening while fitting a generalized linear model. SVM-RFE is a supervised learning model that can be used to analyze data in classification and regression analysis. Random forest is an integrated learning method that produces predictions without considerable bias by training with numerous decision trees. The LASSO regression analysis was carried out using the "glmnet" package in R software (4.2.2). Random forest analysis was performed using the "randomForest" package. SVM-RFE analysis was performed with the "e1071" package.

### Regulation mechanism prediction

Regulatory miRNAs of hub genes were predicted using TarBase v8.0, and transcription factors (TFs) were predicted using the ENCODE database on the NetworkAnalyst platform. Co-expression networks of mRNAs with predicted TFs and miRNAs were constructed using the Cytoscape software (v3.9.1).

### Validation of hub genes

In the GSE129166 dataset, the expression of hub genes was confirmed by the Student’s t-test. The groups were divided into high- and low-expression groups according to the median expression of hub genes in the GSE21374 dataset. The "survival" package was used to investigate survival differences between the high- and low-expression hub genes. Significance was set at *p* < 0.05.

## Results

### Screening of renal IRI and TCMR Co-expression modules

**Fig 2:**
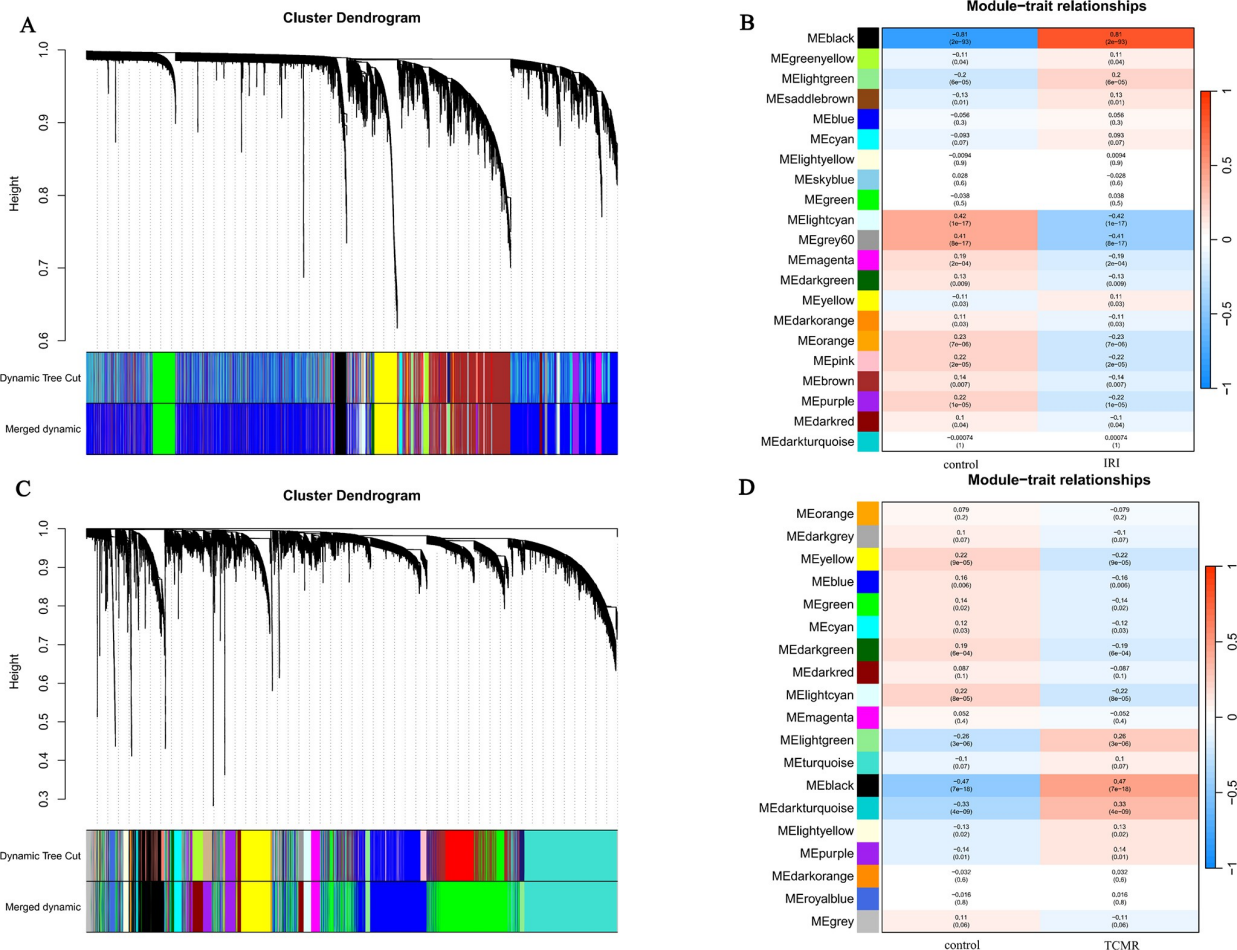
(A) The cluster dendrogram of co-expression genes in renal IRI. (B) Module-trait associations in renal IRI. (C) The cluster dendrogram of co-expression genes in renal TCMR. (D) Module-trait associations in renal TCMR.

In the GSE43974, GSM1075318, GSM1075418, and GSM1075263 datasets were poorly clustered, and the outlier samples were removed. To construct the scale-free network, the scale-free fit index was set at 0.9, and the soft threshold β was 3. Among 21 identified models, module black had the highest correlation with IRI and was the most relevant module (r = 0.81, *p* = 2e-93), with a total of 739 genes (Fig 2A and 2B).

In the GSE36059, GSM880323, GSM880570, GSM880576, GSM880265, GSM880280, GSM880242, GSM880298, GSM880362, and GSM880394 datasets were poorly clustered, and the outlier samples were removed. To construct the scale-free network, the scale-free fit index was set at 0.9, and the soft threshold β was 7. Among 19 identified modules, module black had the highest correlation with TCMR and was the most relevant module (r = 0.47, *p* = 7e-18), with a total of 1,494 genes (Fig 2C and 2D).

### Functional characteristics of shared genes

**Fig 3:**
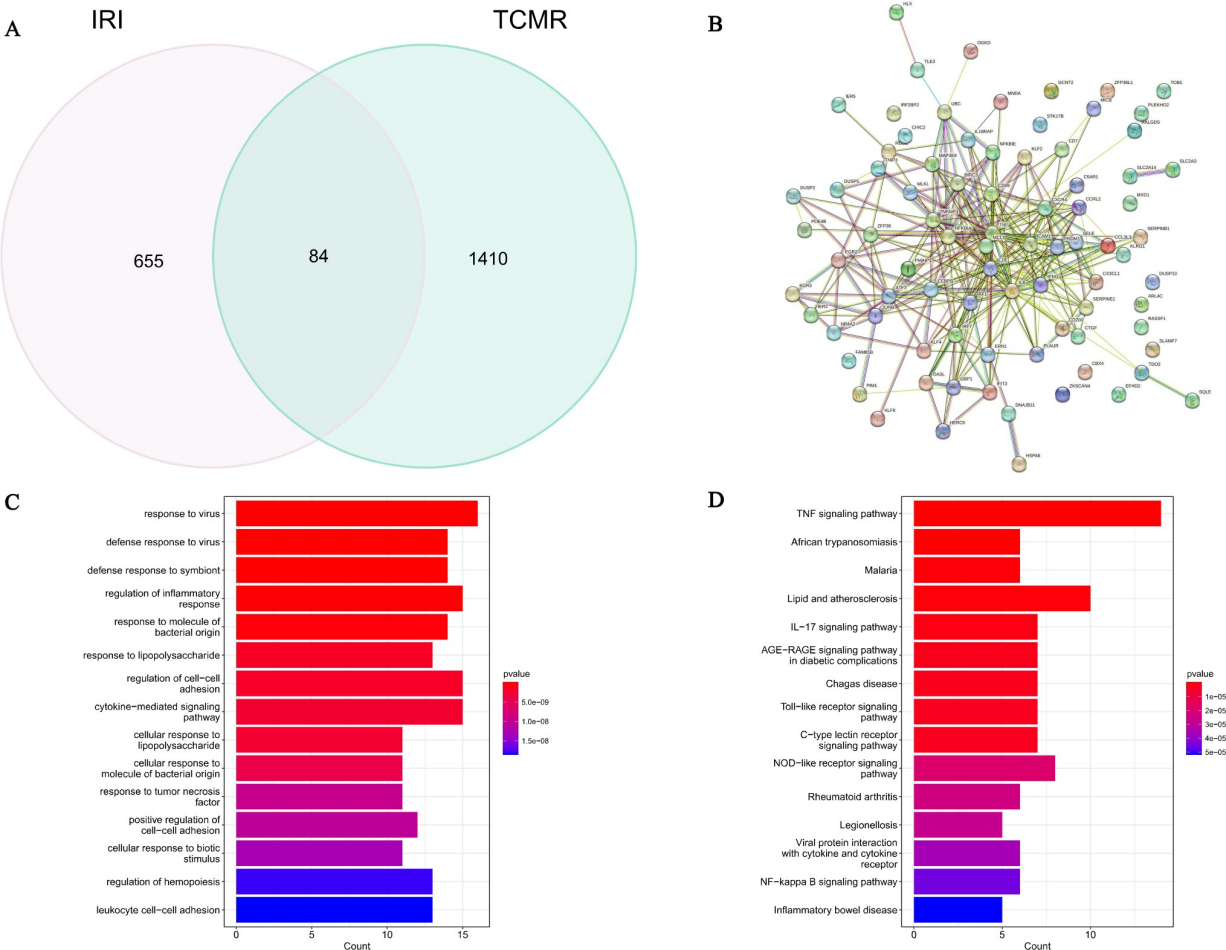
(A) Venn diagram showing shared genes. (B) The PPI network of shared genes. (C) GO analyses of shared genes with a bar plot. (D) KEGG analyses of shared genes with a bar plot.

In the IRI- and TCMR-related modules, 84 shared genes were used to build the PPI network (Fig 3A and 3B). GO analysis showed that these genes were involved in the viral response, inflammatory response regulation, cell-cell adhesion regulation, and cytokine-mediated signaling pathways, among others (Fig 3C). KEGG analysis suggested the shared genes were involved in the TNF signaling pathway, lipid- and atherosclerosis-related pathway, nucleotide oligomerization domain (NOD)-like receptor signaling pathway, interleukin (IL)-17 signaling pathway, and advanced glycation end products (AGE)-receptors for the AGE signaling pathway in diabetic complications (Fig 3D).

### Consensus clustering

**Fig 4:**
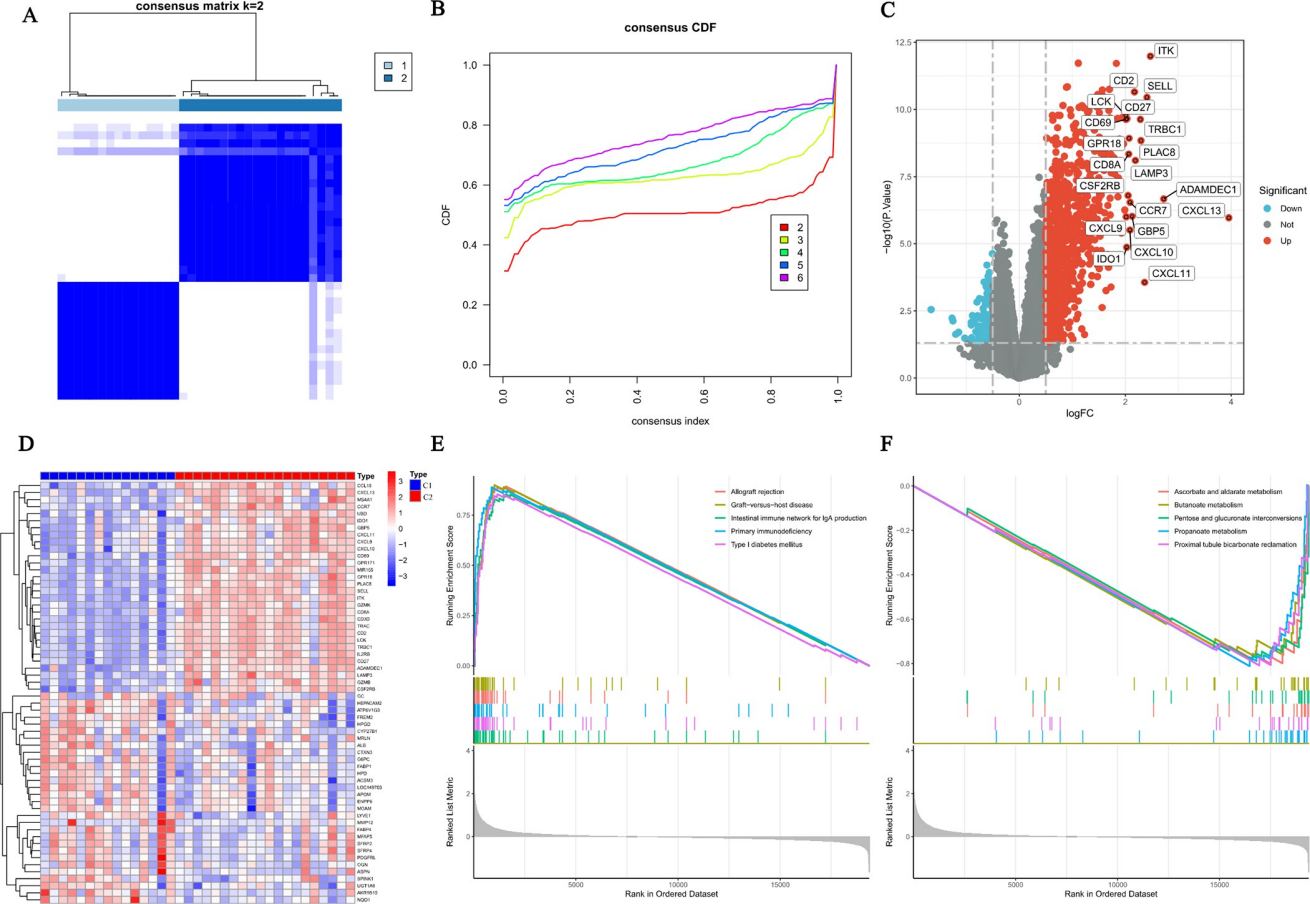
(A) TCMR samples were divided into two clusters when k = 2. (B) Consensus clustering cumulative distribution function for k = 2 to 6. (C) Volcano plot of DEGs between clusters C1 and C2. (D) Heatmap of DEGs between clusters C1 and C2. (E-F) GSEA.

**Fig 5:**
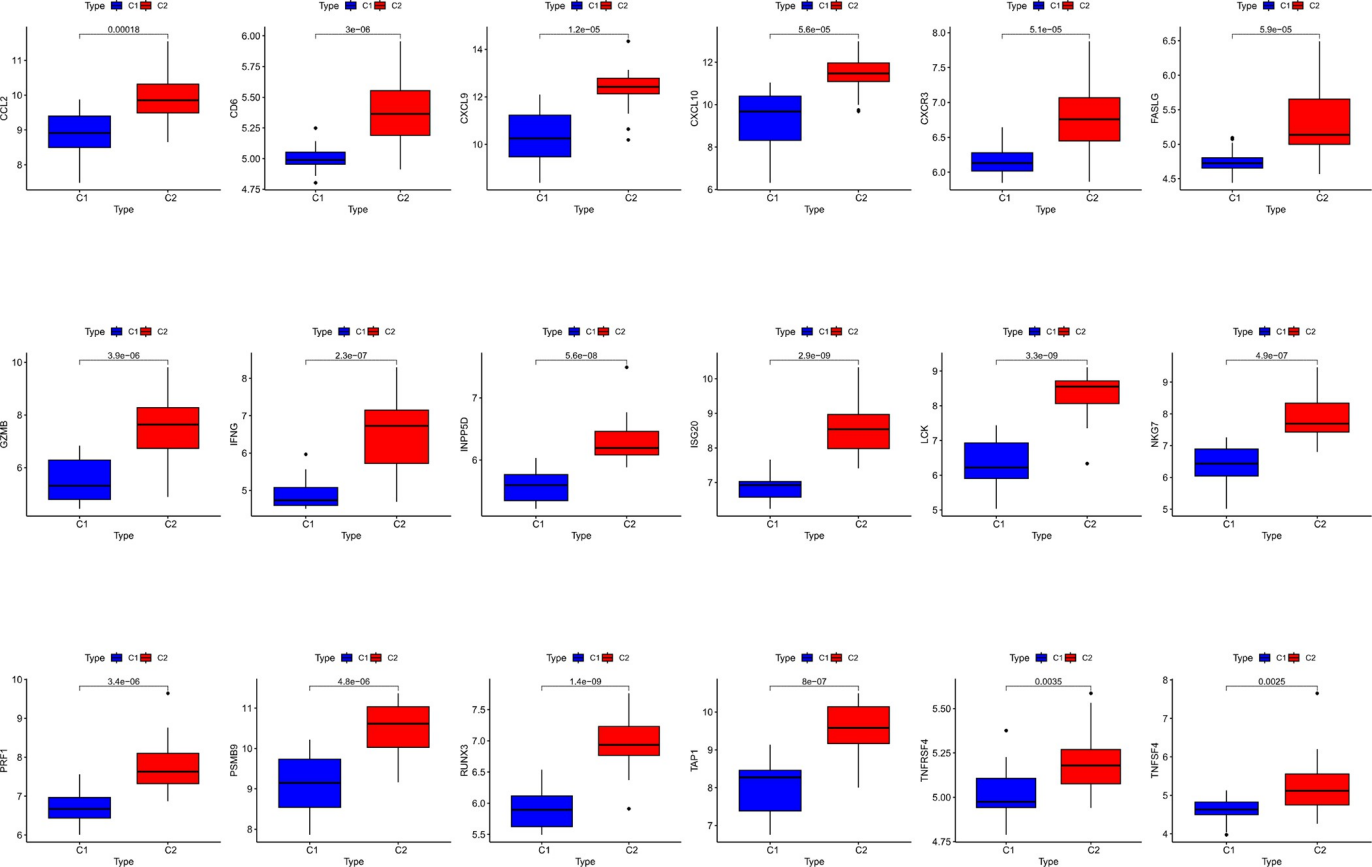
Differential expression of acute rejection markers between C1 and C2 clusters.

In the present study, TCMR was clustered based on gene sharing. Ultimately, k = 2 had the optimal number of clusters, and the TCMR samples were divided into two clusters, C1 (n = 15) and C2 (n = 20) (Fig 4A and 4B). Between clusters C1 and C2, 782 DEGs were identified, of which 743 genes were upregulated and 39 genes were downregulated (Fig 4C and 4D). GSEA revealed differences in the gene set expression of the graft rejection-related pathways between the two clusters (Fig 4E and 4F). Furthermore, the expression of AR markers was elevated in cluster C2, compared with that in C1 (Fig 5).

### Machine learning to screen hub genes

**Fig 6:**
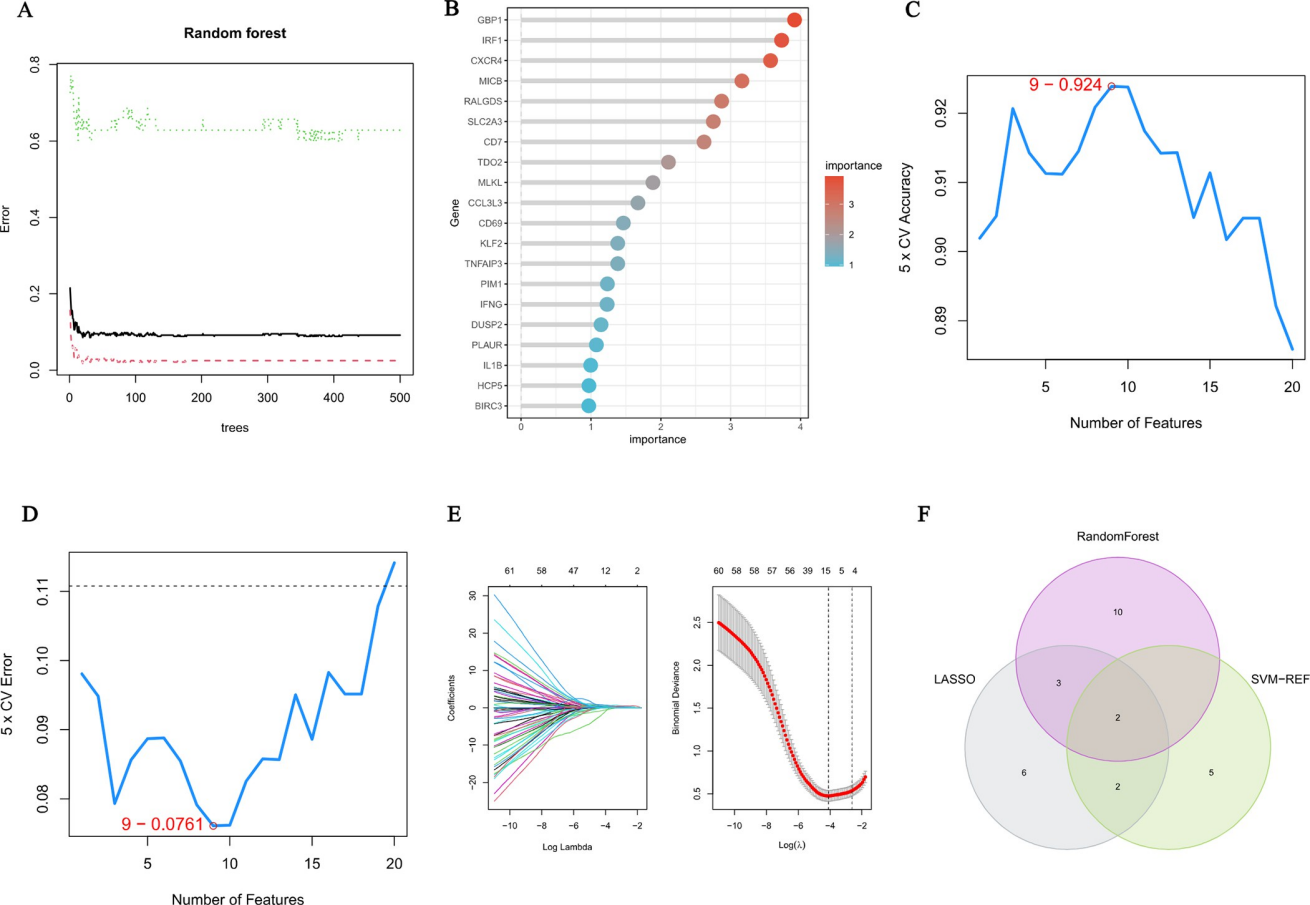
(A-B) Random forest model and gene importance ranking in renal TCMR. (C-D) SVM–RFE model. (E) LASSO regression model. (F) Venn diagram showing the hub genes.

Random forest analysis was performed on shared genes, and the top 15 most important genes were selected (Fig 6A and 6B). The model with nine genes had the lowest error rate and the highest accuracy, according to SVM-RFE analysis (Fig 6C and 6D). LASSO regression model analysis screened 13 genes (Fig 6E). Finally, the intersection of the hub genes screened by the three machine learning methods was used to obtain two genes, CD69 and guanylate-binding protein 1 (GBP1) (Fig 6F).

### Regulation mechanism prediction

**Fig 7:**
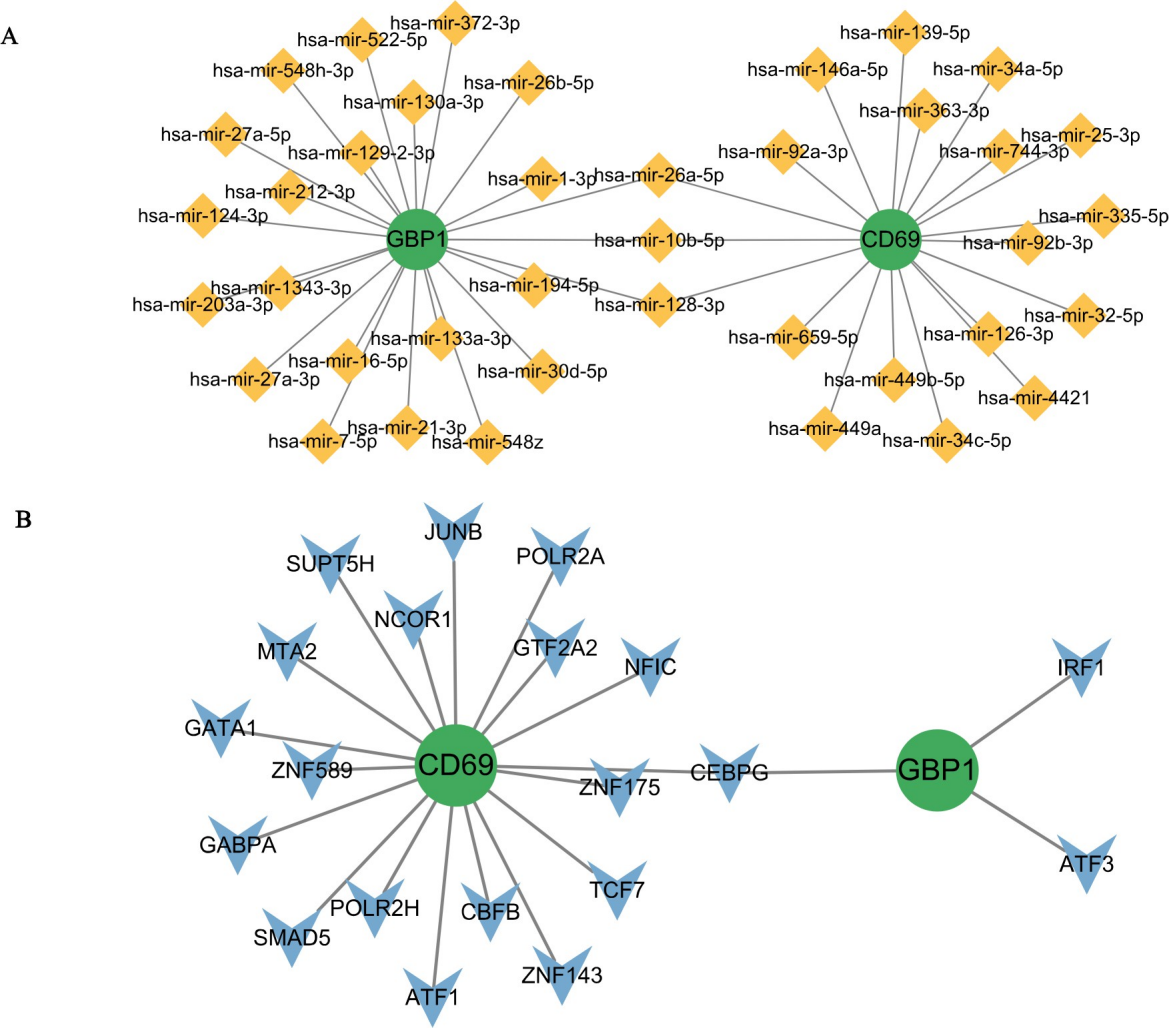
(A) Genes-miRNAs interaction network of GBP1 and CD69. (B) TFs-genes interaction network of GBP1 and CD69.

Regulatory miRNAs common to GBP1 and CD69 included hsa-mir-26a-5p, hsa-mir-128-3p, and hsa-mir-10b-5p, whereas common TFs included CCAAT/enhancer binding protein gamma (C/EBPG) (Fig 7A and 7B).

### Validation of hub genes

**Fig 8:**
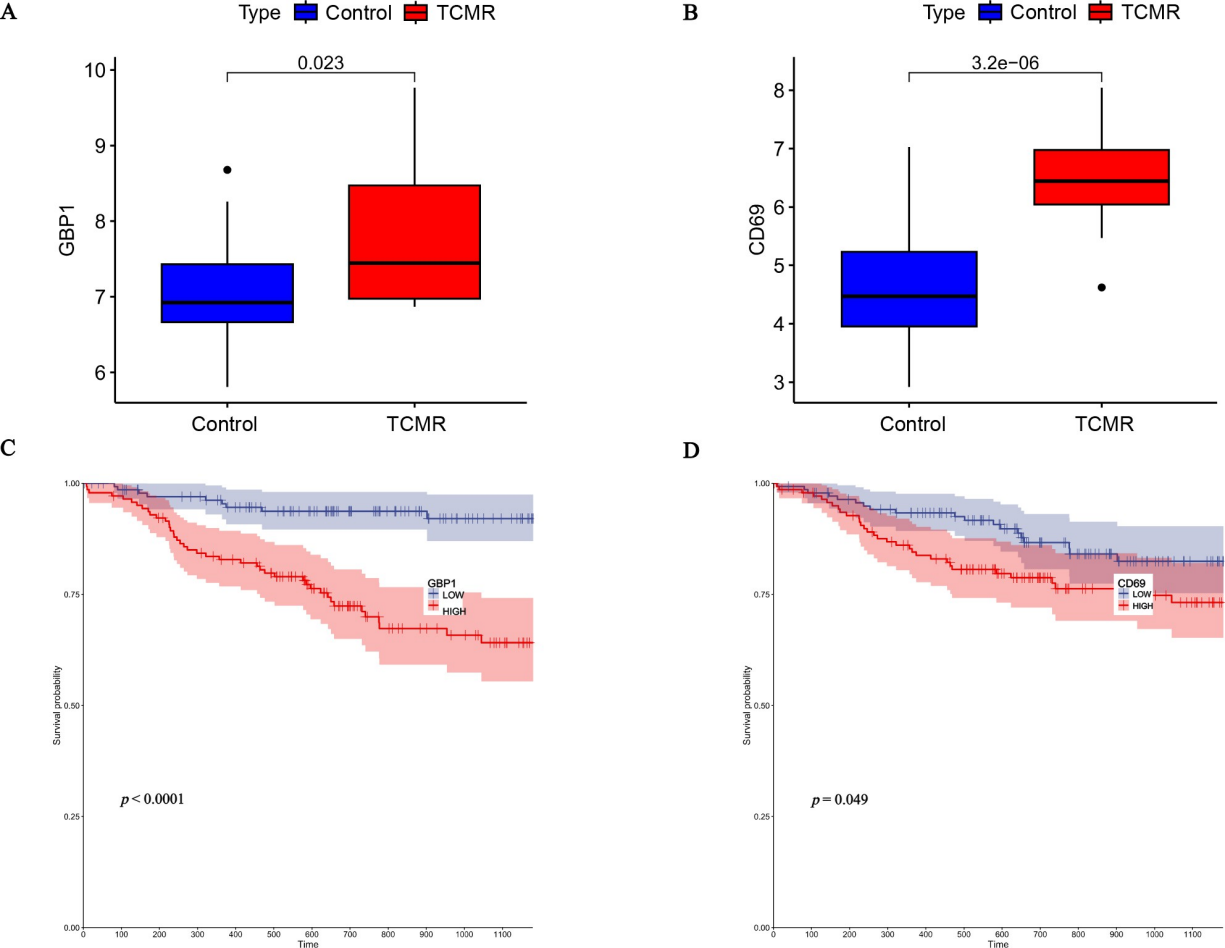
(A) The expression of GBP1 in GSE129166. (B) The expression of CD69 in GSE129166. (C) Survival analysis of KT patients with different GBP1 expression levels. (D) Survival analysis of KT patients with different CD69 expression levels. (The points “·” in the box plot represent outliers).

The expression of the hub genes GBP1 and CD69 was verified in the GSE129166 dataset. The expression levels of GBP1 and CD69 were elevated in the TCMR group (Fig 8A and 8B). Based on the median GBP1 and CD69 expression, the KT samples in the GSE21374 dataset were classified into high- and low-expression groups. Patients who had undergone KT and had high GBP1 and CD69 expression levels experienced lower survival rates (Fig 8C and 8D).

## Discussion

GBP1 is a GTPase primarily induced by interferon-γ (IFN-γ) and involved in processes such as infection and inflammation. Wang *et al*. found that GBP1 knockdown inhibited Bacillus Calmette-Guerin-induced apoptosis in macrophages [12]. In addition, GBP1 protects against dengue virus [13]. Pyroptosis is a significant mechanism of renal IRI [14], and GBP1 is a key protein involved in inflammatory pyroptosis [15]. C/EBP homologous protein/caspase-11 can induce renal tubular pyroptosis [16]. In renal tubular epithelial cells (TECs), Tisp40 can also induce gasdermin D-mediated pyroptosis, and the potential mechanism is related to nuclear factor-κB signaling [17]. In terms of KT, Chauveau *et al*. showed that GBP1 contributes to the diagnosis of antibody-mediated immune rejection [18]. In cardiac transplantation, CD69 has been shown to help with the identification of rejection [19]. CD69 is a marker of tissue-resident memory T cells. Recipient tissue-resident memory T cells are involved in allograft immune responses in KT and are associated with the secretion of TNF-α and IFN-γ [20]. Renal IRI can lead to the long-term infiltration of activated and effector memory T lymphocytes [21]. The continuous exposure of resident memory T cells to antigens leads to allograft rejection [22]. CD69 expression in peripheral blood CD3+ and CD8+ T cells is correlated with AR in patients who have undergone KT [23]. Conversely, it has been suggested that CD69 does not play a role in rejection recognition in KT [24]. Further research is needed to determine whether CD69 and GBP1 expression can be considered markers for KT rejection.

Furthermore, TCMR could be divided into two subgroups based on genes shared by IRI and TCMR, allowing a comparison of the expression of 18 AR markers between the two subgroups [25]. The serum levels of CXCL10, which is widely expressed in chronic allograft nephropathy tissues, help predict graft loss [26]. Ischemia promotes AR by inducing CXCR3 expression, which is associated with inflammatory cell recruitment [27]. Expression levels of granzyme B and Fas ligand were elevated in both the tissue and peripheral blood of patients with AR rejection [28]. The levels of AR markers were higher in the C2 subgroup than in the C1 subgroup. These results suggest that IRI potentially impacts TCMR, and genes shared between IRI and TCMR are important in TCMR.

The inflammatory response plays a significant role in renal IRI and TCMR. Patients with TCMR and glomerulonephritis have poor graft survival [29]. TNF-α induces renal tubular apoptosis in patients with renal IRI, resulting in renal atrophy [30]. Furthermore, TNF-α gene silencing dramatically reduces IRI damage in the liver and kidneys [31]. In KT, TNF-α can target the protein kinase B signaling pathway to induce the epithelial mesenchymal transition in renal TECs, leading to interstitial fibrosis [32]. IL-17 can enhance neutrophil recruitment in kidney IRI [33], and in patients with KT TCMR, increased IL-17 expression has been associated with poorer treatment outcomes [34]. Moreover, during heart transplantation, hyperlipidemia can enhance rejection by increasing IL-17 expression [35]. Activation of the NOD-like receptor protein 3 (NLRP3) inflammasome promotes caspase 1-mediated IL-18 and IL-1β production and is a promising therapeutic target for organ IRI [36]. NLRP3 activation in IRI is associated with mitochondrial reactive oxygen species [37]. In terms of regulatory miRNAs, miR-26a-5p has been shown to protect against brain and myocardial IRI [38] and alleviate sepsis-induced AKI via IL-6 [39]. miR-128-3p targets neuropilin-1 and promotes inflammatory responses in sepsis-induced AKI [40]. However, Xie *et al*. found that the downregulation of miR-128-3p expression in an IRI-AKI model reversed the reduction in apoptosis caused by LINC00963 knockdown [41]. miR-25-3p inhibits IRI-induced apoptosis in renal TECs [42]. miR-27a, whose target is Toll-like receptor 4, inhibits inflammatory responses in renal IRI [43]. Taken together with our results, these pathways provide promising therapeutic targets for the treatment of IRI and TCMR in KT.

The present study had some shortcomings. First, further experimental investigations on the precise molecular pathways implicated by the hub genes (GBP1 and CD69) are required. Second, the clinical data were insufficient to determine the relationship between the hub genes, renal function, and pathological damage. Finally, clinical studies with larger sample sizes are warranted.

## Abbreviations

AGE: advanced glycation end products
AKI: acute kidney injury
AR: acute rejection
C/EBPG: CCAAT/enhancer binding protein gamma
DEGs: differentially expressed genes
FC: fold change
GBP1: guanylate-binding protein 1
GO: gene ontology
GSEA: gene set enrichment analysis
IFN-γ: interferon-γ
IL: interleukin
IRI: ischemia-reperfusion injury
KEGG: Kyoto Encyclopedia of Genes and Genomes
KT: kidney transplantation
LASSO: least absolute shrinkage and selection operator
NLRP3: PP-like receptor protein 3
NOD: nucleotide oligomerization domain
PPI: protein-protein interaction
SVM-RFE: support vector machine recursive feature elimination
TCMR: T cell-mediated rejection
TECs: tubular epithelial cells
TF: transcription factors
TNF: tumor necrosis factor
WGCNA: weighted correlation network analysis

## References

[1] Betjes MGH, Roelen DL, van Agteren M, Kal-van Gestel J. Causes of Kidney Graft Failure in a Cohort of Recipients With a Very Long-Time Follow-Up After Transplantation. Front Med (Lausanne). 2022;9: 842419. doi: 10.3389/fmed.2022.842419 35733857 PMC9207199

[2] Ho J, Okoli GN, Rabbani R, Lam OLT, Reddy VK, Askin N, et al. Effectiveness of T cell-mediated rejection therapy: A systematic review and meta-analysis. Am J Transplant. 2022;22: 772–785. doi: 10.1111/ajt.16907 34860468 PMC9300092

[3] Mizera J, Pilch J, Kamińska D, Krajewska M, Donizy P, Banasik M. Chronic Active T-Cell Mediated Kidney Rejection as a Clinically Significant Type of Allograft Loss? Diagnostics (Basel). 2022;12: 3220. doi: 10.3390/diagnostics12123220 36553226 PMC9777502

[4] Madill-Thomsen KS, Böhmig GA, Bromberg J, Einecke G, Eskandary F, Gupta G, et al. Relating Molecular T Cell-mediated Rejection Activity in Kidney Transplant Biopsies to Time and to Histologic Tubulitis and Atrophy-fibrosis. Transplantation. 2023;107: 1102–1114. doi: 10.1097/TP.0000000000004396 36575574 PMC10125115

[5] Rampersad C, Balshaw R, Gibson IW, Ho J, Shaw J, Karpinski M, et al. The negative impact of T cell-mediated rejection on renal allograft survival in the modern era. Am J Transplant. 2022;22: 761–771. doi: 10.1111/ajt.16883 34717048 PMC9299170

[6] Cherukuri A, Mehta R, Sood P, Hariharan S. Early allograft inflammation and scarring associate with graft dysfunction and poor outcomes in renal transplant recipients with delayed graft function: a prospective single center cohort study. Transpl Int. 2018;31: 1369–1379. doi: 10.1111/tri.13318 30007072

[7] Zhao H, Alam A, Soo AP, George AJT, Ma D. Ischemia-Reperfusion Injury Reduces Long Term Renal Graft Survival: Mechanism and Beyond. EBioMedicine. 2018;28: 31–42. doi: 10.1016/j.ebiom.2018.01.025 29398595 PMC5835570

[8] Nauser CL, Farrar CA, Sacks SH. Complement Recognition Pathways in Renal Transplantation. J Am Soc Nephrol. 2017;28: 2571–2578. doi: 10.1681/ASN.2017010079 28663231 PMC5576943

[9] Li Z, Ludwig N, Thomas K, Mersmann S, Lehmann M, Vestweber D, et al. The Pathogenesis of Ischemia-Reperfusion Induced Acute Kidney Injury Depends on Renal Neutrophil Recruitment Whereas Sepsis-Induced AKI Does Not. Front Immunol. 2022;13: 843782. doi: 10.3389/fimmu.2022.843782 35529856 PMC9069608

[10] Langfelder P, Horvath S. WGCNA: an R package for weighted correlation network analysis. BMC Bioinf. 2008;9: 559. doi: 10.1186/1471-2105-9-559 19114008 PMC2631488

[11] Swift S, Tucker A, Vinciotti V, Martin N, Orengo C, Liu X, et al. Consensus clustering and functional interpretation of gene-expression data. Genome Biol. 2004;5: R94. doi: 10.1186/gb-2004-5-11-r94 15535870 PMC545785

[12] Wang J, Liu Z, Li W, Yu J, Zhang D. Knockdown of GBP1 inhibits BCG-induced apoptosis in macrophage RAW 264.7 cells via p38/JNK pathway. Infect Genet Evol. 2022;97: 105158. doi: 10.1016/j.meegid.2021.105158 34826624

[13] Pan W, Zuo X, Feng T, Shi X, Dai J. Guanylate-binding protein 1 participates in cellular antiviral response to dengue virus. Virol J. 2012;9: 292. doi: 10.1186/1743-422X-9-292 23186538 PMC3520834

[14] Ma N, Lu H, Li N, Ni W, Zhang W, Liu Q, et al. CHOP-mediated Gasdermin E expression promotes pyroptosis, inflammation, and mitochondrial damage in renal ischemia-reperfusion injury. Cell Death Dis. 2024;15: 163. doi: 10.1038/s41419-024-06525-9 38388468 PMC10883957

[15] Johns CE, Galam L. Guanylate Binding Protein 1 (GBP1): A Key Protein in Inflammatory Pyroptosis. Cell Biochem Biophys. 2022;80: 295–299. doi: 10.1007/s12013-021-01056-y 35179710

[16] Yang J-R, Yao F-H, Zhang J-G, Ji Z-Y, Li K-L, Zhan J, et al. Ischemia-reperfusion induces renal tubule pyroptosis via the CHOP-caspase-11 pathway. Am J Physiol Renal Physiol. 2014;306: F75–84. doi: 10.1152/ajprenal.00117.2013 24133119

[17] Xiao C, Zhao H, Zhu H, Zhang Y, Su Q, Zhao F, et al. Tisp40 Induces Tubular Epithelial Cell GSDMD-Mediated Pyroptosis in Renal Ischemia-Reperfusion Injury via NF-κB Signaling. Front Physiol. 2020;11: 906. doi: 10.3389/fphys.2020.00906 32903383 PMC7438479

[18] Chauveau B, Garric A, Di Tommaso S, Raymond A-A, Visentin J, Vermorel A, et al. WARS1, TYMP and GBP1 display a distinctive microcirculation pattern by immunohistochemistry during antibody-mediated rejection in kidney transplantation. Sci Rep. 2022;12: 19094. doi: 10.1038/s41598-022-23078-z 36352007 PMC9646783

[19] Creemers P, Brink J, Wainwright H, Moore K, Shephard E, Kahn D. Evaluation of peripheral blood CD4 and CD8 lymphocyte subsets, CD69 expression and histologic rejection grade as diagnostic markers for the presence of cardiac allograft rejection. Transpl Immunol. 2002;10: 285–292. doi: 10.1016/s0966-3274(02)00072-2 12507400

[20] de Leur K, Dieterich M, Hesselink DA, Corneth OBJ, Dor FJMF, de Graav GN, et al. Characterization of donor and recipient CD8+ tissue-resident memory T cells in transplant nephrectomies. Sci Rep. 2019;9: 5984. doi: 10.1038/s41598-019-42401-9 30979940 PMC6461670

[21] Ascon M, Ascon DB, Liu M, Cheadle C, Sarkar C, Racusen L, et al. Renal ischemia-reperfusion leads to long term infiltration of activated and effector-memory T lymphocytes. Kidney Int. 2009;75: 526–535. doi: 10.1038/ki.2008.602 19092796 PMC2676145

[22] Abou-Daya KI, Tieu R, Zhao D, Rammal R, Sacirbegovic F, Williams AL, et al. Resident memory T cells form during persistent antigen exposure leading to allograft rejection. Sci Immunol. 2021;6: eabc8122. doi: 10.1126/sciimmunol.abc8122 33741656 PMC8103522

[23] Posselt AM, Vincenti F, Bedolli M, Lantz M, Roberts JP, Hirose R. CD69 expression on peripheral CD8 T cells correlates with acute rejection in renal transplant recipients. Transplantation. 2003;76: 190–195. doi: 10.1097/01.TP.0000073614.29680.A8 12865808

[24] Karpinski M, Rush D, Jeffery J, Pochinco D, Milley D, Nickerson P. Heightened peripheral blood lymphocyte CD69 expression is neither sensitive nor specific as a noninvasive diagnostic test for renal allograft rejection. J Am Soc Nephrol. 2003;14: 226–233. doi: 10.1097/01.asn.0000039543.97369.4e 12506155

[25] Salvadori M, Tsalouchos A. Biomarkers in renal transplantation: An updated review. World J Transplant. 2017;7: 161–178. doi: 10.5500/wjt.v7.i3.161 28698834 PMC5487307

[26] Lazzeri E, Rotondi M, Mazzinghi B, Lasagni L, Buonamano A, Rosati A, et al. High CXCL10 expression in rejected kidneys and predictive role of pretransplant serum CXCL10 for acute rejection and chronic allograft nephropathy. Transplantation. 2005;79: 1215–1220. doi: 10.1097/01.tp.0000160759.85080.2e 15880073

[27] Zou X, Song B, Duan J, Hu Z, Cui Z, Gu C. Prolonged ischemia elicits acute allograft rejection involved in CXCR3 activation in rat kidney transplants. Transpl Immunol. 2015;33: 103–109. doi: 10.1016/j.trim.2015.08.001 26303820

[28] Vasconcellos LM, Schachter AD, Zheng XX, Vasconcellos LH, Shapiro M, Harmon WE, et al. Cytotoxic lymphocyte gene expression in peripheral blood leukocytes correlates with rejecting renal allografts. Transplantation. 1998;66: 562–566. doi: 10.1097/00007890-199809150-00002 9753332

[29] Nabokow A, Dobronravov VA, Khrabrova M, Gröne H-J, Gröne E, Hallensleben M, et al. Long-term kidney allograft survival in patients with transplant glomerulitis. Transplantation. 2015;99: 331–339. doi: 10.1097/TP.0000000000000606 25594551

[30] Adachi T, Sugiyama N, Yagita H, Yokoyama T. Renal atrophy after ischemia-reperfusion injury depends on massive tubular apoptosis induced by TNFα in the later phase. Med Mol Morphol. 2014;47: 213–223. doi: 10.1007/s00795-013-0067-3 24407718

[31] Hernandez-Alejandro R, Zhang X, Croome KP, Zheng X, Parfitt J, Chen D, et al. Reduction of liver ischemia reperfusion injury by silencing of TNF-α gene with shRNA. J Surg Res. 2012;176: 614–620. doi: 10.1016/j.jss.2011.10.004 22221603

[32] Zhao C, Xu Z, Wang Z, Suo C, Tao J, Han Z, et al. Role of tumor necrosis factor-α in epithelial-to-mesenchymal transition in transplanted kidney cells in recipients with chronic allograft dysfunction. Gene. 2018;642: 483–490. doi: 10.1016/j.gene.2017.11.059 29174387

[33] Zhang J, Li Q, Zou Y-R, Wu S-K, Lu X-H, Li G-S, et al. HMGB1-TLR4-IL-23-IL-17A axis accelerates renal ischemia-reperfusion injury via the recruitment and migration of neutrophils. Int Immunopharmacol. 2021;94: 107433. doi: 10.1016/j.intimp.2021.107433 33592404

[34] Matignon M, Aissat A, Canoui-Poitrine F, Grondin C, Pilon C, Desvaux D, et al. Th-17 Alloimmune Responses in Renal Allograft Biopsies From Recipients of Kidney Transplants Using Extended Criteria Donors During Acute T Cell-Mediated Rejection. Am J Transplant. 2015;15: 2718–2725. doi: 10.1111/ajt.13304 25989263

[35] Yuan J, Bagley J, Iacomini J. Hyperlipidemia Promotes Anti-Donor Th17 Responses That Accelerate Allograft Rejection. Am J Transplant. 2015;15: 2336–2345. doi: 10.1111/ajt.13350 26079335 PMC5125017

[36] Su X, Liu B, Wang S, Wang Y, Zhang Z, Zhou H, et al. NLRP3 inflammasome: A potential therapeutic target to minimize renal ischemia/reperfusion injury during transplantation. Transpl Immunol. 2022;75: 101718. doi: 10.1016/j.trim.2022.101718 36126906

[37] Wen Y, Liu Y-R, Tang T-T, Pan M-M, Xu S-C, Ma K-L, et al. mROS-TXNIP axis activates NLRP3 inflammasome to mediate renal injury during ischemic AKI. Int J Biochem Cell Biol. 2018;98: 43–53. doi: 10.1016/j.biocel.2018.02.015 29477360

[38] Cheng C, Chen X, Wang Y, Cheng W, Zuo X, Tang W, et al. MSCs‑derived exosomes attenuate ischemia-reperfusion brain injury and inhibit microglia apoptosis might via exosomal miR-26a-5p mediated suppression of CDK6. Mol Med. 2021;27: 67. doi: 10.1186/s10020-021-00324-0 34215174 PMC8254277

[39] Chen Y, Zhou X, Wu Y. The miR-26a-5p/IL-6 axis alleviates sepsis-induced acute kidney injury by inhibiting renal inflammation. Ren Fail. 2022;44: 551–561. doi: 10.1080/0886022X.2022.2056486 35491874 PMC9067948

[40] Wang L, Wang K, Tian Z. miR-128-3p Inhibits NRP1 Expression and Promotes Inflammatory Response to Acute Kidney Injury in Sepsis. Inflammation. 2020;43: 1772–1779. doi: 10.1007/s10753-020-01251-8 32500307

[41] Xie L-B, Chen B, Liao X, Chen Y-F, Yang R, He S-R, et al. LINC00963 targeting miR-128-3p promotes acute kidney injury process by activating JAK2/STAT1 pathway. J Cell Mol Med. 2020;24: 5555–5564. doi: 10.1111/jcmm.15211 32270599 PMC7214170

[42] Zhang Y, Zuo X. miR-25-3p protects renal tubular epithelial cells from apoptosis induced by renal IRI by targeting DKK3. Open Life Sci. 2021;16: 1393–1404. doi: 10.1515/biol-2021-0127 35174294 PMC8812715

[43] Wang Y, Wang D, Jin Z. miR‑27a suppresses TLR4‑induced renal ischemia‑reperfusion injury. Mol Med Rep. 2019;20: 967–976. doi: 10.3892/mmr.2019.10333 31173204 PMC6625210

